# Help, hope and hype: ethical considerations of human microbiome research and applications

**DOI:** 10.1007/s13238-018-0537-4

**Published:** 2018-04-19

**Authors:** Yonghui Ma, Hua Chen, Canhui Lan, Jianlin Ren

**Affiliations:** 1 Centre for Bioethics, Medical College, Xiamen University, Xiamen, 361102 China; 2 Center for Bioethics, School of Marxism, Southern Medical University, Guangzhou, 510515 China; 3 Beijing Rexinchang Biotechnology Research Institute Co. Ltd, Beijing, China; 4 Institute for Human Microbiome Research, Medical College, Xiamen University, Xiamen, 361005 China; 5 Department of Digestive Diseases, Zhongshan Hospital affiliated to Xiamen University, Xiamen, 361004 China

Recent years have witnessed an unprecedented explosion of scientific knowledge and advances in human microbiome research due to the emerging high-throughput molecular technologies. The term human microbiome refers to the population of microorganisms, including bacteria, viruses, fungi and protozoan, and their genetic material that live on and inside the human organisms (skin, mucous membranes, intestinal tract, etc.) (Honey, 2008). A search of the literature at PubMed for the term “microbiome” in the title and abstract illustrates the fast progression of microbiome science. From 2006 to 2010 there were just 304 papers that used the word microbiome in their title and/or abstract, whereas the number has increased to 11,128 from 2011 to 2017. Research on human microbiome, or our second genome, will inevitably bring about dramatic changes in our understanding of ourselves, normalcy, health and illness, and paradigm shift in the management of clinical practice and public health interventions, as well as the production and distribution of commercial products promising health benefits and disease prevention (e.g., individualized diet, probiotics, prebiotics and microbial-based interventions). For example, our commonly used diagnostic criteria for vaginal microbiota wherein the degree of “healthiness” is in part assessed by scoring the abundance of *Lactobacillus* morphotypes, but one study found a quarter of healthy women do not carry *Lactobacillus* in their vagina. This research calls for a better understanding of “normal” and “healthy” vaginal ecosystem that is based on its function, rather than simply on its composition (Ma et al., 2012). Another notable example is the increasing application of the new therapeutic modality of fecal microbiota transplantation (FMT), which runs the risk of being perceived as a panacea for a multitude of illnesses and also the risk of abuse, as increasing websites sprung up advertising home DIY FMT kits as a “self-treatment” salvage (Ma et al., 2017). Extreme care must be taken for microbiome-based interventions to be specific in risk-benefit evaluation and indications for application.

We might need to reconceptualize the human body as an ecosystem and the human being as a superorganism, rather than being a single individual. This re-understanding of ourselves will have important implications on how we address and manage identity, privacy and property issues related to human microbiome, for example, how integral is the microbiome to our conception of self? How does knowledge about microbiome impact what we think it means to be health and disease? To what extent do we own our microbiome given that the source of some microbiome is traditionally considered as waste, e.g., feces? Who can share the benefit when someone’s microbial profile is unique and potentially has commercial value? How should products of microbiome research be regulated and what type of evidence should be required to substantiate health claims for probiotic foods, like yogurt? Should we allow public security agencies to visit Biobanks collecting and storing human microbial samples, which may contain unique and identifiable information of donors?

It is now scientific consensus that the microbiome is important to us and affects our health and disease significantly, however, to what extent is where the debate lies. On the one hand, despite that our knowledge about human microbiome and its impact on human health and disease improves remarkably in the last few years, in fact the strong evidence is still limited; on the other hand, the complex research findings are often presented by social media as “ground breaking”, “attractive”, “exciting” and even “miraculous”, the public though ill-informed but attempt to incorporate the latest “science on human microbiome” into their health decisions while ignoring the long-term and unanticipated risks. There is increasing enthusiasm and effort of manipulating/modifying individual microbiome in pursuing a “healthy” microbiome, but nobody knows what defines a healthy microbiome. And, it’s entirely possible that there isn’t one, or there may be many healthy microbial profiles. Manipulating individual’s microbiome in the hope of achieving better health should not be merely viewed as a technical or medical problem, which also has ethical implications as the changes may affect the surrounding community or society.

It is important to consider the above ethical and social issues early on in human microbiome research to be proactive and prepared in facing challenges. In this article we provide a state-of-the-art overview of the ethical challenges that relate to the broader ethical, legal and social aspects of human microbiome research as well as clinical applications. We focused on (1) human microbiome and personal identity; (2) risks, safety and privacy; (3) informed consent in microbiome-based interventions, e.g., fecal microbiota transplantation and “vaginal seeding”; (4) biobanks; (5) commercialization and hype; (6) public health issues. Throughout the discussion, we also provide some recommendations to the healthy and sustainable progression of human microbiome research. These ethical and social problems must be addressed as part of a successful regulation response to emerging studies and translations of this area.

## HUMAN MICROBIOME AND PERSONAL IDENTITY

Our most familiar question related to personal identity is: who am I or who we are? With the knowledge generated from human microbiome research, if half of our cells are not *Homo Sapiens* cells, what does it mean to be an individual human being? Typically, we draw a distinction between environmental and genetic factors in understanding human traits and the development of disease. Traditionally, the microbial communities around us would be considered as environmental, rather than genetic factors. But with recent findings from human microbiome research that the microbiome co-evolved with human host genome, perhaps we need to reconsider our symbiotic microbiome as “a part of us” than as “a part of the environment”. Some scientific question can also be raised in relation to the question of biological identity, as to how stable and unique is an individual’s microbiome? Is there a core microbiome for human being or particular groups sharing specific commonalities? How permanent changes to the human microbiome might be and whether changes could be transmitted to offspring.

Many commentators agreed on the view that we should think of human as a superorganism comprised of the human body plus the collection of microbes that inhabit the human body. However, commentators discussed the notion of identity issues in human microbiome from different perspectives. According to Gligorov et al., individual and commonsense conceptions of personal identity and self could be affected by the popularization of the human microbiome projects, “the features of our microbiome are features of ourselves” (Gligorov et al., 2013). However, from a philosophical perspective, they also pointed out that numerical criteria for personal identity over time will not be significantly affected by discoveries related to human microbiome. In other words, individual’s psychology or social identity that is most likely to remain the same over time even though his microbiome has been significantly changed, e.g., through fecal microbiota transplantation. In contrast, from an ecological and evolutionary perspective, Dethlefsen et al. believed human microbiome have dramatic implications for how we should understand human being: “the shared evolutionary fate of humans and their symbiotic bacteria has selected for mutualistic interactions that are essential for human health, and ecological or genetic changes that uncouple this shared fate can result in disease” (Dethlefsen et al., 2007). Rhodes resonates with this view, as she asserted that “our coexistence with the microbiome tells us that human evolution is not just human history” (Rhodes, 2013). Nobel Laureate Lederberg believed the combined human-microbiome self is more dynamic and more interactive than we are used to think of ourselves as being (Lederberg, 2006). Finally, from the perspective of individuality and selfness, it has been proposed that the single *Homo Sapiens* is not in fact the real biological individual because the real biological individual is a super-individual defined as the sum of the organism and its microbiome (Hutter et al., 2015).

Outside of philosophy, “personal identity” usually refers to certain properties to which a person feels a special sense of attachment or ownership. Someone’s personal identity in this sense consists of those features she takes to “define her as a person” or “make her the person she is”. There have been some research findings suggest the potential of microbiome-related data to identify group affiliation and more personal information with relation to ethnicity, nationality, race, and even social-economic status. Furthermore, microbes may provide a view of human ancestry. Microbes that constitute human microbiota not only coevolve with humans and maintain complex interactions with hosts, but also can be vertically transmitted. For example, it is suggested that *Helicobacter pylori* (HP) could be employed as a marker of ancestry and migrations (Dominguez-Bello and Blaser, 2011), which means people who share a particular strain of *HP* may have the same ancestor. However, there is considerable skepticism regarding such over-simplified categorization, because it probably neglects the complexity of human microbiome and may bring about new forms of stereotype and stigma.

## RISKS, SAFETY AND PRIVACY

Most research involves some degree of physical, social, or psychological risk. Research ethics requires that the risk of harm introduced by research participation must be balanced against the anticipated social benefits. Emanuel et al. present that studies must have a favorable risk-benefit ratio, which means researchers must examine all of the kinds of burdens involved and compare them to possible benefit and ensure that the benefits outweigh the risks (Emanuel et al., 2000). Research on human microbiome is fraught with numerous unanswered microbiological, clinical, and social questions, therefore balancing possible risks and benefits sometimes very difficult, if not impossible. Particular caution needs to be taken in the context of clinical applications of microbial research.

For participants in microbial research, most human microbiome samples will be gathered through noninvasive or minimally invasive means, for example, only include skin/brushes, oral swabs, saliva collection, nasal swabs, vaginal swabs and fecal self-collection. However, invasive sampling by endoscopy to collect the microbiome of the gut may add a minimal additional risk to conduct the research (McGuire et al., 2008). Because the risks of most human microbiome research and biobanks are often negligible, they involve only the lowest measure of “minimal risk” as defined in many regulations. Rhodes et al. propose a new conception and category of risk, *de minimis* risk, to appropriately describe the risks in the context of human microbiome research, as they explained “it entails a degree of risk so low that harms are nominal and unlikely” (Rhodes et al., 2011). However, as we gain more understanding of variation in the microbiota that inhabit different parts of the body, as well as the advantages of deep versus minimally invasive sampling, sampling techniques and associated risk-benefit assessments may change.

### Case study: from fecal microbiota transplantation to “vaginal seeding”

In the clinical application of human microbiome-based interventions, the risks are far more uncertain and complex. We employ FMT as an illustration to demonstrate these uncertainties and risks, and raise the caution about labeling some groups as “risky”.

FMT is the delivery of large amounts of intestinal microbiota from a prescreened healthy donor into the intestinal tract of a patient (Ma et al., 2017; Borody and Campbell, 2012). It is currently the most effective therapy for recurrent *Clostridium difficile* infection (CDI) and is also a potential treatment for a variety of diseases beyond digestive tract (Surawicz et al., 2013; Cammarota et al., 2014). Although current research suggests FMT is safe and no serious adverse events have been reported, there are many areas in which evidence is lacking. The most notable concern is the potential transmission of anxiety and depression. Evidences are accumulating that indicate the gut microbiota interacts with the central nervous system (CNS) and can influence brain function and behaviour (Cryan and Dinan, 2012). Increasing studies of microbial transplant research in mice models showed gut microbiota influencing stress and anxiety-related behavior. Fecal microbiota transplantation of germ-free mice with “depression microbiota” derived from human patients with depression resulted in depression-like behavior compared with colonization with “healthy microbiota” derived from healthy control individuals (Zheng et al., 2016). Some scholars even worried that “by transferring mood-/mind-altering microbes, FMT may carry the possibility of altering a person’s personality and identity (positively and negatively)” (Ma et al., 2017), this also raised the philosophical implication on personal identity and autonomy.

Another case is the practice of “vaginal seeding”. This technique involves swabbing a mother’s vagina fluids and transferring it to the mouth, eyes and skin of a newborn baby born by caesarean section. It’s claimed that this practice may stimulate microbiome development similarly to babies born naturally—and protect it from health issues later in life, e.g., allergies and asthma. The theory of vaginal seeding is to allow for proper colonization of the fetal gut and, therefore, reduce the subsequent risk of asthma, atopic disease and immune disorders (Seeding, 2017). This practice started with a 2010 paper by Dominguez-Bello et al., who conducted a study on 21 babies and found babies who were seeded with the gauze absorbed with vaginal microbiome of mother had a microbiome closer to a baby born vaginally than those born via C-section (Dominguez-Bello et al., 2010). This intervention has soon attracted enthusiasm from expectant mothers asking for advice on the procedure and performing this intervention on themselves. As many as 90% of Danish obstetricians and gynecologists said that they had been asked about it by prospective parents. Sometimes, the interested parents will practice this technique with their own hands when it is difficult to find a doctor willing to perform the procedure.

However, this procedure carries serious potential risk of transferring pathogenic organisms from the woman to the neonate. A recent article published by the American College of Obstetrics and Gynecology has ruled the procedure “unnecessary” and in some cases, “downright dangerous” (Seeding, 2017). The article stated the risk of performing vaginal seeding includes undiagnosed *C. trachomatis*, *N. gonorrhea*, human papilloma virus, group A streptococci, and herpes simplex virus infections, among others, at the time of delivery that could result in neonatal infection that may otherwise been avoided by cesarean delivery without seeding. It is concluded that “vaginal seeding should not be performed outside the context of an institutional review board-approved research protocol until adequate data regarding the safety and benefit of the process become available”. Similarly, an editorial in the BMJ also takes a skeptical stance: “it might seem reasonable to perform this simple and cheap procedure, even without clear evidence of benefit, but only if we can be sure that it is safe” (Cunnington et al., 2016). Ironically, the hypothesis that baby born under different delivery mode may have significant different microbiome has been challenged. A recent study (Chu et al., 2017) involving more than 160 pregnant women and their babies found that the mode of delivery, vaginal versus cesarean section, did not affect the infants’ microbiome composition. In addition, there is concern about the long-term risks on children by manipulating their microbiota, as found that there is a critical period in infancy and early childhood, when the immune system is still developing and is in part shaped by the gut microbiota, during which the manipulation of gut microbiota has its greatest impact on the health and brain causing lifelong changes (Rodriguez et al., 2015; Schulfer and Blaser, 2015).

### Psychological and social risk

Apart from the above known and unknown physical risks, there are also psychological and social risks associated with participating in human microbiome research, which warrant serious consideration. Due to the sensitive nature of microbial data (both clinical and genetic), risks arising in microbial research may lead to negative consequences for participants regarding privacy breaches.

Psychological risk includes reactions like anxiety and depression, as well as the disclosure of clinically relevant information or an incidental finding (IF) about the research participants. To illustrate, during screening, potential participants will be tested for HIV and other sexually transmitted infections while a positive test result will exclude the individual from participation and cause depression on him (McGuire et al., 2008). In this situation, the researchers may want to inform the participants of the positive results but there is a possibility that the individual may not want to know (McGuire et al., 2008). In many societies, HIV and related people are strongly discriminated and marginalized. Further questions are also relevant about whether researchers have an obligation to report such findings to public health authorities, or individuals who may be infected. Furthermore, it’s been widely discussed that analyzing microbiome sample from research may also reveal disease susceptibility, e.g., an increased risk of obesity or colon cancer, based on current studies that have shown a correlation between intestinal microbiota and diseases. This particular information may have positive implications for the subject’s current behavior, and informing this discovery to the participants would allow him to change lifestyle, diet and other factors that might reduce the risk of diseases (McGuire et al., 2008). This seems even more feasible as microbial intervention and microbial data are more clinically actionable than human genetic data. However, there are also concerns about the possibility that sharing these information with participants could produce psychological distress or depression (Schwab et al., 2013). After all, at this early stage, knowledge of the human microbiome is too premature to make any conclusions regarding disease risks and susceptibility, or how the microbiome should be manipulated to restore health. Therefore, as suggested by Ma et al., researchers, scientists, and social media need to take a precautionary approach towards the validity of any conclusions in this kind to avoid labeling some people as risky to some diseases, especially in societies where there are social stigma against mental illness and sexual transmitted diseases (Ma et al., 2017).

Information obtained from human microbiome research may also subject the participant to social risks and privacy breaches (Ma et al., 2017; Chuong et al., 2017; Hawkins and O’Doherty, 2011). Information of human microbial profile when combined with human genomic and medical information, this could generate unprecedented personal-revealing information of a new magnitude, not only the knowledge of disease susceptibility, but also as travel experience, sexual practice, as well as consumption of alcohol and drugs. The disclosure of these informations could potentially make some individual ineligible for health, disability, or life insurance coverage or employment application. Historically, we are familiar that some medical problems will carry social stigma and discrimination and victims may be excluded from the mainstream society (Schwab et al., 2013). Moreover, since recent advances in human microbiome research have demonstrated that “personal microbiomes contain enough distinguishing features to identify an individual over time” (Franzosa et al., 2015), namely, each human carries unique personalized “microbial fingerprints” or “microbial cloud” (Meadow et al., 2015). The possibility that individual could be identified simply by analyzing his microbiome (with or without his human DNA) opens up a powerful new analytical dimension for forensic investigations or law enforcement (Tridico et al., 2014; Hampton-Marcell et al., 2017; Schmedes et al., 2016). Although this could help augment existing trace evidence options for forensic researchers, it might also cause disastrous social harms to the person involved, e.g., deportation or incarceration. However, since the stability of microbial fingerprints over time is unknown, it is unclear how reliable they are uniquely identifying for individuals, and what might be known about an individual with only a sample of his microbiota.

### Protection of privacy

Identifying the above concerns about the physical, psychological, and social risks raised by human microbiome research is not intended to impede the progress of this area, but to highlight the importance that protection should be provided for participants and data arising from research. It is important to examine whether the existing regulations and protections are sufficient to protect people from privacy breaches and whether modifications are needed. It is suggested by some researchers that the current protections of genetic information should be amended and extended to apply to human microbial samples (Hawkins and O’Doherty, 2011; Gligorov, 2013), for example, the Genetic Information Nondiscrimination Act (GINA) and Health Insurance Portability and Accountability Act (HIPAA) in the USA and Personal Information Protection and Electronic Documents Act (PIPEDA) in Canada. They call for human microbiome samples to be treated with the same privacy safeguards as human tissue samples, based on the possibility to identify individuals. Yet, in many non-Western countries, the area of human microbiome research, including some clinical interventions like FMT, remains largely unregulated. This lack of regulation could increase the risks of microbiome-based intervention misuse and the exploitation of vulnerable patients/participants by unethical research and commercial agents. There is a clear and urgent need to establish regulatory mechanism to address the unique risks of human microbiome research in different social and legal contexts.

## INFORMED CONSENT

Both in clinical practice and biomedical research on human subjects, obtaining informed consent have become a well-established moral and legal obligation and requirement since 20th century. Informed consent is also the cornerstone principle of research ethics (Emanuel et al., 2000). Consent is considered fully informed when a competent patient or research subject to whom full disclosures have been made and who understands fully all that has been disclosed, voluntarily consents to treatment or participation on this basis (Rosamond Rhodes et al., 2013). The concept of respect for autonomy explains the centrality of informed consent in the ethical practice of research (Ruth, 1986). Informed consent and respect for autonomy of people begins with the Nuremberg Code, which was in response to the cruelty of Nazi experiments, stipulating “The voluntary consent of the human subject is absolutely essential” (Ruth, 1986). The tradition of respecting autonomy and informed consent was given primacy in the following international ethics guidelines, including World Medical Associate’s Declaration of Helsinki, the Belmont Report, and the International Ethical Guidelines for Biomedical Research Involving Human Subjects issued by Council for International Organizations of Medical Sciences (CIOMS).

In biomedical research, discussions of consent primarily focused on how to ensure valid consent when the nature of research and the related potential risks and benefits of participation are unknown. This issue has been acknowledged by many commentators as equally challenging in human microbiome research (Ma et al., 2017; Rhodes, 2016; McGuire et al., 2008; McGuire et al., 2012): although it is already common practice to collect and store microbiome samples from people as well as their personal medical data, given our limited knowledge about the human microbiome, it is almost impossible for researchers to provide a full explanation about specific research related information using microbiome samples. Moreover, in human microbiome biobanking research, microbial samples are collected from multiple body sites and will be linked with human host’s data, but we are unable to predict the future research and the potential risks associated with that research. These facts raise an important ethical issue as whether participants should be permitted to give general consent to unknown future research and whether this form of consent is “valid” and “voluntary”. Some argues that it is acceptable that participants are informed the uncertainty inherent in conducting microbial research as a condition of participation, while research advances and new risks and benefits discovered, changes to the consent process are necessary (McGuire et al., 2008). But this practice faces similar critique as in human biobank regarding the erosion of participant’s protection of autonomy in decision-making (Caulfield, 2007). General consent is not genuine valid consent because it deprives the opportunity to withdraw if the nature of future research is not consistent with the values and priorities of participants. Another unique problem in human microbiome relates to the stability and dynamic nature of microbiome samples. It is questionable about the value and necessity of re-consent in microbiome biobanking research, which may be unnecessary and unfeasible as microbiome sample collected may not be identifiable to the same person over time.

### Shift the primacy of informed consent: from autonomy to “solidarity”

However, there are also voices to shift the primacy of informed consent and the autonomy of participants in research ethics, and call for a new perspective on the ethical conduct of human research. As Rhodes and Schwab et al. argued, the research ethics traditionally focus on autonomy and informed consent because they were in response to clinical trials which may expose subjects to possibly serious harm. However, the current human microbiome research and personalized medicine require broad participation to provide samples with incomplete knowledge of the research (Juengst et al., 2012) while often exposing participants to only “*de minimis*” risks (as we explained in last section) of physical harm (Rhodes, 2016; Schwab et al., 2013). Meanwhile, these research efforts promised to promote health are worthy of pursuing (Rhodes, 2016; Rosamond Rhodes et al., 2013). A careful re-evaluation of the benefit-to-harm ratio is mandatory. Therefore, we should advocate for a shift from concentrating on autonomy of participates, to the principle of “solidarity” which entails the obligation of individuals to contribute to the enterprise of biomedicine research.

### The vulnerability of patient/research participants

Apart from the difficulty of uncertainties of information related to human microbiome research, subject’s decision making capacity, voluntariness, and vulnerability also demand serious consideration, especially in clinical trials research and innovative therapies. For example, in FMT, patients with acute or refractory inflammatory bowel disease (IBD) are particularly “vulnerable due to their healthcare experiences with ineffective therapies and subsequent poor quality of life, both of which may increase the propensity to make healthcare decisions based solely on desperation” (Rubin et al., 2014). Other sources of vulnerability is the IBD patient’s susceptibility to nutritional deficiency, as well as higher risks of developing mental and psychological conditions, such as depression and anxiety (Bannaga and Selinger, 2015). Based on these studies, it is questioned that the autonomy of IBD patients as an FMT target population may be “compromised by their stress and desperation, affecting their ability to give informed consent” (Ma et al., 2017). They further argued that “their capacity to be informed may also be affected by a diminished ability to appropriately process information about risk” and these vulnerable IBD patients/subjects are susceptible to “deception or inducement” from some overhyped claims for the therapeutic effects of FMT.

### How much should be included in informed consent documents?

McGuire raised a practical issue concerning the effectiveness of informed consent documents used in Human Microbiome Project as they were too long and complex for the average research subject to understand. As she explained, these documents “not only did they have to address consent for the storage and use of biological specimens and the attendant privacy risks that get implicated in genomic research, but they also had to address the physical risks associated with the complicated nature of sampling from 15–18 body sites” (McGuire et al., 2012). Although there are proposed changes to shorten the documents, it remains unclear, what would constitute a set of minimum appropriate information which should be included for informed consent, in order for patient/subject to make educated, autonomous decisions regarding their treatment or participation in research. As in the case of FMT, guidance is needed on whether we should outline every current potential issue to the patient (including the possibility of transmission of depression and anxiety?), or whether only what we currently understand to be principal concerns associated with FMT.

## BIOBANKS

### Overview of microbiome biobanks

With the development of human microbiome research, it is inevitable that a growing number of biobanks will include a collection of microbiota specimens required for genomic studies. These human microbiota biobanks and the associated genetic information may become a valuable health resource. Similar to biobanks storing human tissue samples, e.g., the UK Biobank, the types of specimens collected and the associated clinical information stored vary with the purpose and scope of the human microbiome biobank, as well as the nature of the research (Ma et al., 2017). For example, American Gut is an open-source, community-driven effort to characterize the microbial diversity of the American public and to understand how diet and lifestyle may contribute to health through each person’s gut microbes. Disease specific biobanks like the cystic fibrosis biobank in Canada aims to facilitate sample and data sharing for research into the link between disease progression and microbial dynamics in the lungs of pediatric and adult patients (Chuong et al., 2017). The Multi-Omic Microbiome Study-Pregnancy Initiative (MOMS-PI) is a collaborative project based at Settle Children’s and funded by the NIH to understand the impact of the vaginal microbiome on pregnancy, pregnancy-related complications and the impact on the fetal microbiome. Using multi-“omics” technologies, it was aimed to collect samples from a cohort of 2000 women throughout the course of pregnancy to explore how the microbiome impacts risk for preterm birth and the temporal dynamics of the pregnancy microbiome.

### Stool banks

Among various banks, perhaps the most contentious and promising biobank in recent years would be “stool banks”, which have shown considerable potential in treating patients with intestinal tract disorders, especially those infected with CDI (van Nood et al., 2013; Costello et al., 2016), as well as contributing to translational research of human gut microbiome (Bolan et al., 2016). A stool bank has been defined by the US FDA as “an establishment that collects, prepares, and stores FMT products for distribution to other medical institutions, healthcare providers, or other entities for therapeutic or clinical research”. The benefit of stool bank is straightforward, as our recent questionnaire survey on gastroenterologist clinicians in China found that the majority of the respondents were greatly in favor of the establishment of a fecal microbiota bank as it “not only shifts the burden of contact with stools, but also avoids the privacy problems” (Ma et al., 2017). So far, a growing number of stool banks have been established globally, OpenBiome was the first one launched in 2012 in Massachusetts, USA, and has provided stool for 13,000 FMT in USA and six other countries. There are also stool banks organized by other research institutes and clinics, for example, AdvancingBio in the USA, the Taymount Clinic in the UK, the Netherlands Donor Faeces Bank (NDFB), Melbourne FMT in Australia, Asian Microbiota Bank in Hongkong, and the Chinese FMT Bank in China. These banks are fundamentally important for medicine. On the one hand, knowledge gained from these banks, donors and patients will help to optimize the therapeutic use of stool samples; on the other, using high-throughput techniques will also make it feasible to identify and analyze all the microorganisms associated with the human gut together with host genetics, as well as to understand how these contribute to changes in the gut microbial ecosystem (Ma et al., 2017).

However, due to the symbiotic relationship with the host of the sample, human microbiome biobanks present practical, social, and ethical problems, some of which are distinct from those of biobanks storing human tissue samples. For example, how to determine and select healthy donors, how to manage and interpret the unprecedented amount of biological data when the relevance to disease is unknown, how to externalize and collaborate with other banks and clinics nationally and internationally, should volunteers be rewarded financially for giving stool samples to incentivize stool donation (Bolan et al., 2016; Terveer et al., 2017; Paramsothy et al., 2015). Ethical issues in biobank are mainly focused on privacy, informed consent, ownership of samples and information, secondary use of biological specimens, benefit sharing, rules and governance (Cambon-Thomsen, 2004; Bledsoe, 2017). We will mainly review two issues below:

### Ownership

Hawkins and O’Doherty raised the question “who owns your poop” and argued that this ownership problem may become even more complex with microbial research (Hawkins and O’Doherty, 2011): “not only because feces has traditionally been considered to be waste, but because of the ambiguous relationship between the genomes of commensal bacteria found in fecal matter and human identity”. They further explained the difficulty of ownership: on the one hand, microbial genomes are clearly not a part of the human genome and so should not be considered in any way a component of ‘being human’; on the other hand, human genome has co-evolved to its present state with bacterial genomes and we require this symbiotic relationship for the maintenance of our health. Microbial genomes we carry may be “almost as personal as our own genome” (Hawkins and O’Doherty, 2011). Although we have not realistically encounter ownership problem in biobanks storing human microbial samples so far, the issue of ownership can be reflected by many parallel cases in other human research. The case of Henrietta Lacks is a good illustration: an African American woman whose tumor cells were used in medical research without her knowledge or consent. This case also shows that ownership issues are closely connected to future benefit sharing. The question of ownership may become even more complex with microbial research as there is still a lack of scientific consensus on whether human microbiome data is uniquely identifying like human genetic data and the stability of the microbiome over time (Ma et al., 2017). It is highly possible that information generated from someone’s fecal sample could not be linked to the individual after years, or when he or she takes antibiotics, or even changes diet.

### Return of results to participants

In biobank studies, there has been much debate on whether research participants have a right to know (or not to know) information (Ravitsky and Wilfond, 2006; Brownsword and Wale, 2017), who should be responsible for informing and explaining to participants, to what extent of the result and information should be disclosed, and what criteria should be adopted for returning research results. Moreover, the issue of validity and clinical utility of the research results is also highly relevant as there is a risk of harm due to the premature or inadequate translation of research results (Burke et al., 2010). Regarding the criteria for returning research results in microbial research, it is suggested that findings that are “analytically valid, reveal an established and substantial risk of a serious health condition, and are clinically actionable” should be returned to participants (Chuong et al., 2017). In human microbial research, these issues “becomes more complicated as the validity, reliability, relevance, and clinical significance of human microbial data are largely uncertain” (Ma et al., 2017). In contrast, unlike human genome which is relatively static throughout life, human microbiome is much more dynamic and changeable, which gives rise to great excitement among scientific community as well as general public to modulate and intervene our microbiome (Chuong et al., 2017).

## HYPE AND COMMERCIALIZATION

The human microbiome research has raised hopes that new scientific knowledge could be used for better diagnostic tests or new interventions to modulate our microbiota to cure disease. But there is also hype sometimes mixed with hope, which is dangerous and risky, and may discredit the value of microbial research in the long run.

### Hype in industry

Increasing volume of studies have found a wide variety of diseases are associated with changes in the human microbiome, in particular the gut. These findings are often (mis)presented and (mis)interpreted by popular social media as “exciting” and “attractive”, which create excitement and enthusiasm among the general public (and some clinicians). People are led to believe that microbiome is connected to all human organs and diseases, as well as high expectations that knowledge of the human microbiome will solve all medical problems (Hanage, 2014; Bik, 2016). Companies offer personalized analysis of the microbial content of fecal samples, promising consumers enlightening information and providing targeted individual diet and nutritional product. The industry of “probiotics”, despite still lack of evidence and regulation in most countries (Harrison et al., 2015), is a rapid growing business, with annual global sales of products reaching 36.6 billion dollars in 2015. Many of the products such as yogurts containing probiotics/prebiotics are popularly sold in the supermarkets but vary widely in manufacturing process, strain and dose of microorganisms, and quality control (Slashinski et al., 2012). The commercialization of the scientific value of “good” bacteria, and some pharmaceutical companies may cite the findings and claim health benefits even though their commercial products may not have the same strains and formulations as those tested in published studies. These claims (and hypes) echo the enthusiasm and excitement expressed when the first draft of the human genetic sequence published in 2001. This was viewed as a huge step in biomedicine as claimed human genome sequence would allow researchers to “uncover the hereditary factors in virtually every disease”, and “adapt therapies to the individual patient” (Collins, 1999). However, the promised advances have been slow and the human genome project was not more than another step in the research of human genetics. On the tenth anniversary of the publication of the draft, an editorial in Nature noted that its promise is “still to be fulfilled” (Best is yet to come, 2011). We should be cautious of the risk that microbiome initiative might encounter the same backlash.

### Hype in scientific research

It is important to separate the hype from hope and health, but it is not always easy as some of this over-enthusiastic interpretation of data has been found in scientific papers as well. Noting the disturbing trends, Hanage (Hanage, 2014) warned that “microbiomics risks being drowned in a tsunami of its own hype” and called for those interpreting research should ask five crucial questions: 1) can experiments detect differences that matter? 2) does the study show causation or just correlation? 3) what is the mechanism? 4) how much do experiments reflect reality? 5) could anything else explain the results? He also reminded “the history of science is replete with examples of exciting new fields that promised a gold rush of medicines and health insights but required skepticism and years of slogging to deliver”. Similarly, Bik highlighted the issue “animals are not humans” (Bik, 2016). Results obtained in animal models have to be interpreted with caution, as which are not necessarily applicable to humans. Mice and humans are quite different with respect to body, sizes, diet, gut anatomy and functions (Nguyen et al. 2015).

### Hype is dangerous

In a health oriented consumer culture, commercialization of health products is very powerful. For instance, scientific debate over the effectiveness and safety of food supplements and probiotics doesn’t affect their popularity (Senok et al., 2005; Nettleton, 2013). In an interview with scientists and researchers, Slashinski et al. found the commercialization of human microbiome research opens the door for “commercialized intervention”, or the proliferation of commercial products that claim maintenance or restoration of good health with the use of good bacteria. They warned under the current paradigm of health that encourages individual responsibility and marketing strategies appealing to empowering effects of dietary supplements, these hypes have ethical implications of “therapeutic misconception”. The hype of microbiome-based interventions is particularly dangerous, for instance, individuals who are led to believe the promised health benefit of probiotics may not be informed, or ignore, the potential risks of probiotics and put their safety at risk, especially for those with immune deficiency and young children. Sharp et al. argued that for patients who are to make informed choices about the use of probiotics, they must consider the above safety-related issues that have not been well characterised, otherwise, they may have incomplete appreciation of the risks and cannot make well-informed choices about probiotic therapies (Sharp et al., 2009).

Apart from risk to individuals, hype could also cause backlash effect for legitimate treatment. As Ma et al. argued, if patient’s DIY FMT leads to unfavorable consequences, disappointed individuals may lose confidence in FMT and perceive it as “quackery” and sharing their feelings on social media outlets, which will significantly discredit FMT (Ma et al., 2017). Finally, it is important to note that overhyped rhetoric will unconsciously affect not only patients, but also clinicians and researchers. In a survey on perspectives of Chinese clinician towards FMT, Ma et al. found clinicians generally have negative attitudes towards some social media exaggerating and mystifying the effects of FMT as “magic” and “miraculous”, which may mislead patients. However, they also found one third of these clinicians chose “more natural and organic” as the reason for recommending FMT to their patients, which may also be misleading in itself, as “natural” and “organic” are never value-free words but rather commonly understood as the meaning of “safe” and “less risk”, somehow “healthier” (Ma et al., 2017).

To guard against hype, concerted efforts need to be made among researchers, pharmaceuticals, social media and journalists, and regulatory bodies: the scientific community needs to develop better experimental methods to evaluate conclusions; pharmaceuticals and manufactures must be responsible for ensuring the safety of their products and being truthful about the actual health benefits; social media and popular science writers must stop exaggerating and selectively reporting results; and regulatory body such as FDA must take responsibility to ensure the safety or effectiveness of products throughout manufacture, marketing and distribution. There is an urgent need to find a balance between the marketplace, scientific research and the public’s health.

## PUBLIC HEALTH IMPLICATIONS

Historically, microbe-related diseases have posed significant threat to public health. Efforts to protect and promote public health require disease surveillance, tracking, and data collection on disease outbreaks and deaths. Research on human microbiome has transformed our traditional “common knowledge” and societal practices, from perceiving that all bacteria are harmful and should be eliminated, to realizing that most our dwelling bacteria are “friendly” to us, not “enemies”, and that with over-consumption of antimicrobial products, we are actually harming ourselves and the effect may be long lasting. Studies have found our gut microbiome’s adaptation to a modern diet, rich in simple carbohydrates and dairy products, may play important role in the development and perpetuation of world obesity epidemic (Walter and Ley, 2011; Sanmiguel et al., 2015).

Human microbiomes do not exist in isolation from each other, they are constantly evolving and influenced by environmental factors such as antibiotic use, diet, and the microbiomes of family and other community members (O’Doherty et al., 2016). Drawn from debates surround public health and ethical implications of microbiome research, we concentrate on three points: First, as O’Doherty pointed out, microbiome technologies designed to have an effect on individual level may have important and unanticipated consequences on family, community, and public health levels (O’Doherty et al., 2016). We should be aware of the fact that people are both potential vectors and victims of disease (Francis et al., 2005). There are increasing evidence that microbes can be transmitted and shared between people who come in close contact, family members, and even members of the same sports (Turnbaugh et al., 2009; Schwarz et al., 2008). The possibility that transmission of microbes may permanently affect microbial profiles (especially for children) raises important implications on autonomy. This means individuals may have their microbiomes altered as a result of other people’s health decisions, which may against their wishes (or even without their knowledge) in ways that are detrimental to them (O’Doherty et al., 2016).

Second, it is important to note the risk of cross-contamination of microbiomes between different species. In their book *Beasts of the Earth*: *Animals, Humans and Disease*, Torrey and Yolken showed an unintended consequence of a poultry industry decision to increase efficiency and profit by creating chickens with a variation in their micorbiome so as to eliminate a threat to chickens. Unknowingly, it introduced a risk to human consumers (Torrey and Yolken, 2005; Rosamond Rhodes et al., 2013). This case is a clear example of the complex interaction among the microbiome of a different species and how efforts to produce benefits may have unintended consequences (Rosamond Rhodes et al., 2013).

Third, from public health perspective, microbiome-based interventions may be designed to target and promote community and public health. For example, if a particular probiotic being developed to target eradicating of *HP* (a bacterium that may lead to stomach cancer), this probiotic may be introduced into the food system or water supply, it may be possible to reduce rates and suffering from stomach cancer (Lu et al., 2016; Oh et al., 2016). However, there has also been evidence that the decline of HP may be associated to the increase in diseases of the esophagus caner, and people without HP are more likely to develop hay fever, asthma and skin allergies in childhood (Chen and Blaser, 2007). This case highlights the complexity of the function and role of bacteria; they may be both harmful and helpful. Public health interventions normally limit the liberty and decision making of individuals. How public health concerns can justify limiting individual liberty, how knowledge from microbiome research can be used to design sound public health policies, and how to evaluate the promising public benefit against the potential costs, are all important questions to answer before the implementation of public policies. Moreover, public interventions that affect the microbiome of infants and children, as well as other vulnerable groups, need to be precautionary by properly evaluating unintended long-term consequences.

In addition, human microbiome research also opens up opportunities to address the global challenges of infectious disease and antibiotic resistance. For example, studies have suggested that antibiotic use could be avoided if fecal microbiota transplant were used for the treatment of recurrent CDI as an earlier intervention rather than as a last resort (Ma et al., 2017) and this would reduce economic costs compared with standard antibiotic therapy (Merlo et al., 2016; Varier et al., 2015). Balskus pointed out that “a better understanding of the mechanisms underlying colonization resistance and other microbiome-pathogen interactions may reveal new strategies for treating or preventing infections” (Balskus, 2016). Tosh and McDonald suggested that infection control programs need to be reframed “to capitalize on the expanding understanding of the protective role of the microbiome” and “to preserve and reestablish a harmonious endogenous microbiome” (Tosh and McDonald, 2012). Appropriate public health policy involves a complex assessment of risks, harms and benefits that affect the entire population.

## CONCLUSIONS

Human microbiome research, together with new technologies, has the potential to increase our understanding of how health and disease are affected by the complex relationship between human, our dwelling microbiome, and the environment. The advancement of this area will inevitably shift the paradigm of managing clinical practice and public health interventions. As with other innovative research, the ethical, legal, and social implications (ELSI) are complex and worthy of careful consideration by researchers, healthcare professionals, and regulatory agencies alike. In this paper, we provided a state-of-the-art overview of these challenges and focused on the debates over six core issues: (1) personal identity; (2) risks, safety, and privacy; (3) informed consent; (4) biobanks; (5) commercialization and hype; and (6) public health implications. These issues have been encountered in other research context, e.g. Human Genome Project, but they are further complicated by the unique and distinctive challenges raised by microbiome research, for example, the concern about “microbial fingerprint” and related privacy breaches. We suggest that ethical framework and regulations on human microbiome research is urgently needed with respect of informed consent, privacy, return of result, commercialization, and data protection.

With our expanding knowledge of microbial mechanism, our stereotypical notion of “good” versus “bad” microbes needs to be changed. Some promising probiotics may have unanticipated side effect for particular groups, while traditionally perceived harmful microbes have been found to have novel and potentially unanticipated benefits. Whether a microbe is helpful or harmful is highly dependent on complex host and microbial factors. We need to be cautious with microbiome manipulation interventions on their broad effects on the host beyond the conditions they are designed to treat. More carefully designed trials and long-term follow up studies should be developed.

Discussions in this paper reflect the value of incorporating ELSI into the human microbiome research from its beginning. ELSI researchers need to be involved in the research initiative’s conception and consulted on the study design, protocol development, and informed consent process. This engagement is vital to the success of research. We need to be proactive and prepared in addressing ethical and social challenges instead of reacting to harm afterwards with rush decisions. We believe only by doing so, a meaningful, responsible, and sustainable collaborative endeavor can be fostered to better regulate and guide the development of human microbiome initiative.
